# Maternal heat stress reduces body and organ growth in calves: Relationship to immune status

**DOI:** 10.3168/jdsc.2021-0098

**Published:** 2021-06-03

**Authors:** B.M.S. Ahmed, U. Younas, T.O. Asar, A.P.A. Monteiro, M.J. Hayen, S. Tao, G.E. Dahl

**Affiliations:** 1 Department of Animal Sciences, University of Florida, Gainesville 32608; 2 Department of Animal and Dairy Science, University of Georgia, Tifton 31793

## Abstract

Late-gestation heat stress of dairy cows reduces fetal growth and influences postnatal performance and immune status of the offspring. Our first objective was to evaluate the effect of in utero heat stress on overall fetal and organ growth, particularly organs associated with immune function. The second objective was to examine the cellular mechanism of altered passive immunity in neonatal bull calves after in utero heat stress. Specifically, we examined the rate of apoptosis of intestinal cells early in life, as it is associated with gut closure. Dams were dried off approximately 45 d before expected calving and randomly assigned to 1 of 2 treatments: heat stress (HT) or cooling (CL). During the dry period all cows were housed under shade in a freestall barn, where the pen for CL cows was equipped with active cooling, including water soakers and fans, whereas the pen for HT cows had no soakers or fans. Using rectal temperature and respiration rate as indicators, heat stress was severe. Average rectal temperature in HT cows was 39.3°C compared with 39.0°C in CL cows, and HT cows had a respiration rate of 66.7 breaths/min compared with 43.2 breaths/min for CL cows. Bull calves (n = 30) were immediately separated from their dams at birth, weighed, and then killed before colostrum feeding (n = 5/treatment; d 0) or at 1 or 2 d of age following colostrum feeding (n = 5/treatment per day). After slaughter, the small intestine was removed and weighed, and samples from the jejunum were fixed for immunohistochemistry. Birth weight of bulls from HT dams was 1.1 kg lower than that of bulls from CL dams. Thymus, spleen, and heart weights of HT bulls were lower relative to those of CL bulls, whereas liver weight of HT bulls tended to be lower relative to that of CL bulls. Jejunal cell apoptosis decreased with age in both HT and CL calves after birth, mirroring gut closure. However, in utero heat stress increased the apoptotic rate in the jejunum, particularly at birth. We conclude that the chronic exposure to heat strain of HT compared with CL dams in late gestation significantly affected fetal growth and immune tissue development, which may be associated with reduced immune function in early life. Also, late-gestation heat stress increased calves' intestinal apoptosis in the first 2 d of life, which might explain the decreased IgG uptake and limited passive immune competence observed in previous studies.

## Highlights

• In utero heat stress reduces growth relative to calves born to cooled dams.
• Immune organ growth is further compromised beyond whole body growth.
• Jejunal enterocyte apoptosis is accelerated at birth after in utero heat stress.

## Graphical Abstract

**Figure:**
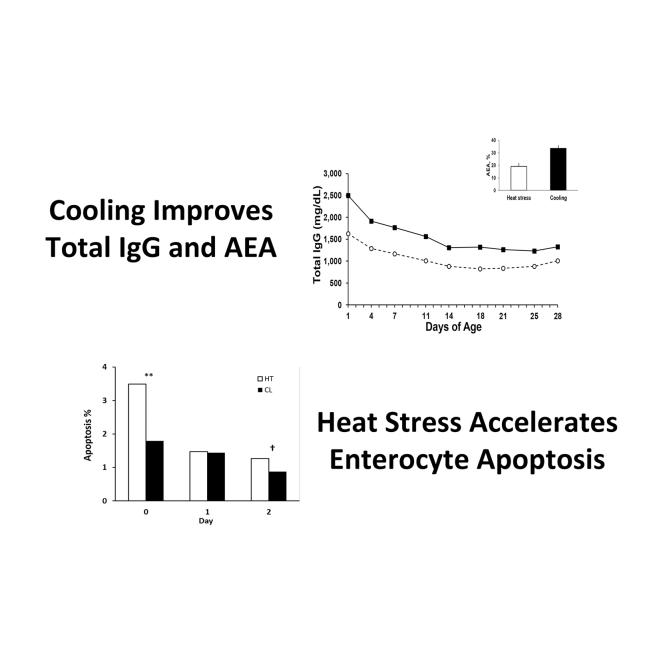
**Summary:** Following in utero heat stress in late gestation, calves have lower circulating immunoglobulin concentrations relative to calves gestated in dams that received heat stress abatement. As illustrated in the top panel, IgG is lower from the first day of life and that reduction persists through the first month of life, which is a challenge to calf health. Total IgG ingested from colostrum did not differ, indicating that the calf's capacity for absorption of IgG was compromised. Uptake of IgG in the first days of life is associated with turnover (i.e., apoptosis) of the initial population of enterocytes in the small intestine, which are replaced by enterocytes that form tight junctions and limit movement of large molecules such as IgG. We tested the hypothesis that in utero heat stress increased the rate of enterocyte turnover thereby reducing the capacity for IgG uptake from colostrum. The lower panel illustrates the relative acceleration of enterocyte apoptosis, consistent with more rapid closure of the gut and reduced passive transfer. AEA = apparent efficiency of absorption.

Dams exposed to heat stress in late gestation have calves with lower birth weights, which reflects compromised fetal development (Tao et al., 2011, 2012; Tao and Dahl, 2013). In utero heat stress not only hinders fetal growth but also influences the postnatal physiology, such as immune function and metabolic adaptation, of the offspring (Monteiro et al., 2014, 2016; Dahl et al., 2016). Studies indicate that calves born to and fed with colostrum from cows exposed to heat stress during late gestation have compromised passive immunity, as measured by apparent efficiency of IgG absorption (**AEA**) and cell-mediated immune function, compared with calves born to cows that were cooled when dry (Tao et al., 2012). Further, the lower AEA associated with in utero heat stress is a direct effect on the calf rather than an effect of altered colostrum (Monteiro et al., 2014). However, the mechanism causing the reduced AEA with in utero heat stress is unknown.

Uptake of macromolecules such as immunoglobulins across the intestine is transient and limited to the first 24 to 36 h of life (Staley and Bush, 1985; Sangild, 2003). Jochims et al. (1994) suggested that enterocyte populations are rapidly replaced early in life and that gut closure is due to mature enterocytes replacing the initial population that actively absorb immunoglobulins. Indeed, Castro-Alonso et al. (2008) observed that the rate of enterocyte apoptosis in goat kids was consistent with the rate of gut closure, whereby increased apoptosis was paralleled by lower immunoglobulin absorption. Based on our previous observations regarding in utero heat stress reducing immunoglobulin uptake and overall fetal growth (Monteiro et al., 2014) as well as preliminary evidence that heat stress altered immune organ development (Ahmed et al., 2016), we hypothesized that in utero heat stress would decrease organ weight and alter the normal trajectory of gut closure relative to CL. The first objective of the present study was to determine the effect of in utero heat stress on overall fetal growth and organ weights, particularly those organs associated with immune function. The second objective was to examine the cellular mechanisms of altered passive immunity in neonatal bull calves after in utero heat stress during late gestation, specifically by examining the rate of apoptosis of intestinal cells early in life.

Animal use was approved by the University of Florida Institutional Animal Care and Use Committee. The experiment was conducted during the summer over 2 consecutive years (2014 and 2015) at the University of Florida Dairy Unit (Hague, FL; 29.77°N, 82.42°W). All treatments of cows and the resultant calves were assigned randomly and balanced across both years. During the dry period, all cows were housed under shade in a freestall barn, where the pen for cooled (**CL**) cows was equipped with active cooling, including water soakers over the manger and fans over the manger and stalls, whereas the pen for heat-stressed (**HT**) cows had no soakers or fans. The average temperature-humidity index of the pens housing the pregnant cows was 77.9 and 77.6 for CL and HT, respectively, in 2014 and 77.6 and 77.9 for CL and HT, respectively, in 2015. Rectal temperature was measured using a digital thermometer every day at 1400 h (GLA M700; GLA Agricultural Electronics). Respiration rate was determined by visual observations of flank movements for 1 min, and measurements were made every day at 1400 h. Feed and water were available ad libitum for all cows at all times. All calves were raised postnatally under identical management in stalls under shade.

Cows were cooled (n = 40) or heat stressed (n = 40) during an approximately 45-d dry period that represented approximately the final 45 d of pregnancy. After birth, all bull calves were immediately separated from their dams and weighed. Bull calves (n = 30) were killed using a captive bolt to stun the animal followed by exsanguination at birth without colostrum feeding (n = 5/treatment) and at 1 and 2 d of age following colostrum feeding (n = 5/treatment per day). To avoid confounding effects of maternal colostrum, pooled good-quality colostrum (>50 g of IgG/L; 3.8 L) was fed by esophageal tube according to University of Florida Dairy Unit standard operating procedures within 4 h after birth to bulls slaughtered on 1 and 2 d of age. Calves killed on 1 and 2 d of age received 4 L of pasteurized whole milk daily. After slaughter, the wet weight of the small intestine and other organs, including the thymus, spleen, pancreas, liver, heart, kidney, and lung, was recorded. The small intestine was removed and washed with PBS, and a tissue sample from the jejunum was collected for immunohistochemistry.

Jejunal samples for immunohistochemical analysis were fixed in 4% neutral formalin overnight at 4°C and then transferred to 70% ethanol until embedding. Samples were dehydrated and embedded in paraffin (MBI Cell and Tissue Analysis Core, University of Florida, Gainesville) following standard protocols and sectioned at 4 µm onto slides coated with poly-l-lysine. To investigate the relationship of enterocyte apoptosis and compromised passive immunity after late-gestation heat stress, terminal deoxynucleotidyl transferase dUTP nick end labeling was performed on sectioned jejunal samples to estimate the apoptotic rate of intestinal cells using the same procedure described in Tao et al. (2011). Briefly, after deparaffinization and hydration, samples were digested with 20 μL/mL proteinase K (Ambion; Applied Biosystems Inc.) for 8 min at room temperature (24°C), followed by washing with double-distilled water. Endogenous peroxidase activity was quenched by incubation with 2% H_2_O_2_ in PBS for 10 min. Following incubation in equilibration buffer (ApopTag Plus Peroxidase In Situ Apoptosis kit; Millipore) for 10 min, sections were incubated with terminal deoxynucleotidyl transferase (333 U/mL) and digoxigenin-conjugated nucleotides (Millipore) for 60 min at 37°C. Reactions were stopped by stop/wash buffer and then washing extensively with PBS. Labeled DNA was detected with anti-digoxigenin peroxidase (ApopTag Plus Peroxidase In Situ Apoptosis kit, Millipore) for 30 min at room temperature followed by colorimetric detection with 3,3′-diaminobenzidine. Sections were counterstained with methyl green, dehydrated, and mounted, and counting was done under a 40× objective on a Nikon Optiphot microscope (Nikon Corp.). Apoptosis was estimated by counting the number of positive cells in a field (stained brown, indicating cell apoptosis) to calculate the percentage of positive cells out of total cells in 6 different sections per slide per calf.

Rectal temperature, respiration rate, birth weight, organ weight, and immunohistochemistry data were analyzed by least squares ANOVA with PROC GLM of SAS (version 9.4; SAS Institute Inc.). Cow or calf was considered a random variable, and treatment, time, and treatment × time were considered fixed effects. In addition, if there was a treatment × time interaction, the pdiff mean separation test was used to compare HT and CL at each individual time. Data are presented as least squares means ± standard error of the mean. Significance was declared at *P* < 0.05, whereas tendencies were declared at *P* < 0.15. Heat stress during the dry period increased cow rectal temperature (HT = 39.3 ± 0.02 vs. CL = 39.0 ± 0.02°C; *P* < 0.01) as well as respiration rate relative to cooling (HT = 66.7 ± 0.48 vs. CL = 43.2 ± 0.48 breaths/min; *P* < 0.01). Therefore, as expected, heat stress during the dry period decreased bull birth weight (HT = 39.3 ± 1.11 vs. CL = 43.8 ± 1.11 kg; *P* < 0.01; Table 1).

**Table 1:** Organ weights of bull calves that were heat stressed or cooled in utero during the final ~45 d of gestation^1^

Organ weight	Treatment (Trt)						SEM	*P*-value		
	Heat stressed			Cooled						
	d 0	d 1	d 2	d 0	d 1	d 2		Trt	Day	Trt × day
BW, kg	38.65	41.64	37.10	44.72	41.64	42.27	1.89	0.02	0.49	0.24
Small intestine, g	983	1,300.9	1,355.4	1,080.4	1,348.2	1,442.8	96.9	0.34	<0.01	0.96
Thymus, g	101.4	126.6	95.2	149.1	132.3	132.5	14.4	0.02	0.54	0.33
Spleen, g	68.6	78.8	79.1	81.4	89.3	110.4	6.9	<0.01	0.03	0.28
Pancreas, g	20.8	27.9	25.6	21.5	25.1	28.3	2.2	0.92	0.02	0.46
Liver, g	722.4	946.6	765.2	878.3	880.8	983	70.4	0.09	0.28	0.13
Heart, g	289.4	303.9	284	327	324.4	336.9	19.6	0.03	0.95	0.71
Kidney, g	157.8	174.3	171.2	164.8	155.6	199.8	11.5	0.56	0.10	0.14
Lung, g	531.3	508.2	470.3	576.9	486	536.9	40.9	0.38	0.33	0.53

1 Calves were killed on 0, 1, and 2 d of age (n = 5/d per treatment).

Proportional with the decrease in BW, in utero heat stress decreased organ weights (Table 1), especially of immune organs such as the thymus and spleen (Table 1; *P* = 0.02 and *P* < 0.01, respectively) as well as the heart (*P* = 0.03). Additionally, in utero heat stress tended to decrease liver weight (*P* = 0.09) compared with in utero CL calves (Table 1). When organ weights were adjusted as percentages of BW, thymus and spleen weights tended to be reduced in HT bulls (*P* = 0.08 and 0.15, respectively). Likewise, there was a slight tendency for a treatment × day effect on liver and kidney weights (*P* = 0.13 and *P* = 0.14, respectively; Table 1), whereby in utero HT calves had lower liver and kidney weights compared with in utero CL calves, particularly at 2 d of age.

Regardless of treatment, jejunal cell apoptosis decreased with day of age of calves, mirroring the time course of gut closure (*P* < 0.01; Figure 1). However, the jejunum of in utero HT calves had a higher percentage of apoptotic cells at birth relative to in utero CL calves (*P* < 0.01; Figure 1). When examined based on whether calves received colostrum, in utero heat stress increased the rate of apoptosis in the jejunum of calves before colostrum feeding (*P* < 0.01; Figure 1).

**Figure 1:**
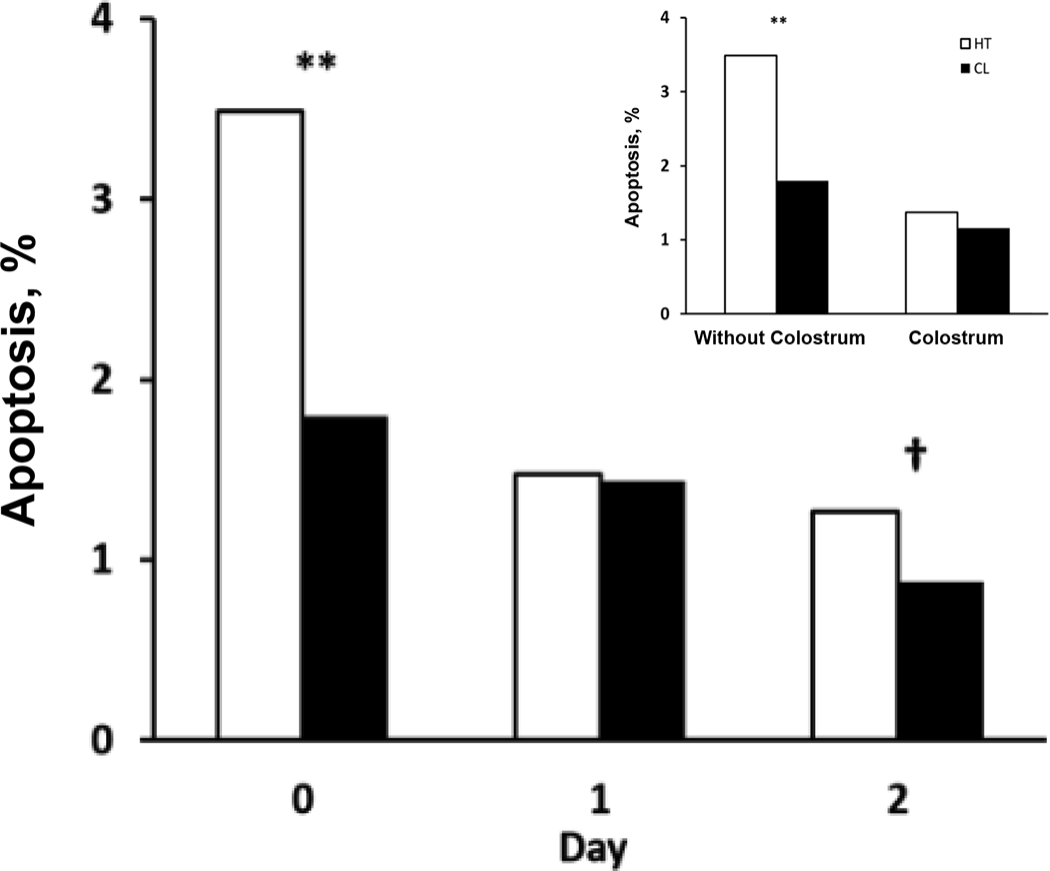
Percentage of apoptotic jejunum tissue samples from bull calves collected early in life following different environmental treatments in utero. Treatments were in utero heat stress (HT; open bars; n = 5/d) and in utero cooled (CL; solid bars; n = 5/d), and days of age were birth (0 d) and 1 and 2 d. The inset represents the apoptotic percentage of bull calves that either were fed colostrum or did not receive colostrum. Calves that did not receive colostrum were killed shortly after birth (d 0), whereas calves receiving colostrum were killed at d 1 and 2 of age. Least squares means of treatment × day (SEM = 0.22) are reported. Effect of treatment (*P* < 0.01), day (*P* < 0.01), and treatment × day interaction (*P* = 0.09). **P < 0.01; †*P* < 0.10.

Elevated rectal temperatures and respiration rates (i.e., 0.3°C and 23.4 breaths/min higher in HT vs. CL) of the dams that did not have access to fans and soakers indicate that heat stress was severe in the current study. Therefore, as expected, we observed a difference between treatments in the birth weight of calves, with HT calves having lower birth weight relative to CL calves. This observation is consistent with previous studies and likely reflects reduced placental function and reduced gestation length in the cows exposed to heat stress in late gestation (Tao et al., 2011, 2012; Monteiro et al., 2014). Indeed, we recently reviewed the effect of late-gestation heat stress on gestation length and found that heat-stressed dams calve an average of 2 d earlier than cooled herdmates (Dado-Senn et al., 2020). Muller et al. (1975) estimated that calves grow between 0.4 and 0.6 kg/d during the last week of gestation; thus, it is unlikely that the 3-kg birth weight differential observed in the present study was due to a shorter gestation length.

Not only were birth weights reduced by in utero heat stress, but individual organ mass was also lower than that in CL calves. In utero heat stress reduced heart weight and tended to decrease liver weight compared with CL calves. Likewise, there was a tendency for treatment × day interaction on liver and kidney weights, whereby in utero HT calves had smaller liver and kidney mass relative to in utero CL calves, particularly at 2 d of age. As heat stress of the dam in late gestation compromises placental and fetal development, fetal heart, liver, and kidney weights are reduced, which is consistent with observations in sheep. For example, Dreiling et al. (1991) reported that lambs from ewes that were chronically heat stressed during gestation had lower birth weight and proportionally lighter organs compared with normothermic control lambs. Further, Limesand et al. (2006) observed a reduction in organ weights in lambs gestated in ewes that were heat stressed from d 40 to 97 of pregnancy, and those morphological changes were associated with significant shifts in metabolism. Thus, the reduction in organ development with heat stress in the present study was consistent with that seen in other ruminants.

Calves, like all young animals, are prone to elevated rates of mortality and morbidity in the neonatal period, and preterm birth may further exacerbate that problem (Flemming and Nielsen, 2001). Early-life losses may result from organ immaturity and dysfunction, notably of the gastrointestinal tract (Sangild et al., 2000) and the immune system (Barrington and Parish, 2001). Because passive uptake of immunoglobulins is the only mechanism of immunoprotection in the bovine, neonatal calves are consequently quite susceptible to insults that might reduce the transfer of colostral immunoglobulins (Barrington and Parish, 2001). As reported, lymphoid Peyer's patches, lymph nodes, and the thymus are the lymphoid tissues in the gut where most lymphocytes are found (Reynolds, 1997). More severe models that result in lower thymus weight also reduce immune function. For example, treatment with dexamethasone caused thymic atrophy, reduced serum IgG, and lowered lymphocyte proliferation in calves (Cantiello et al., 2007). Compared with ad libitum feeding, calves that were malnourished had lower thymus weights and lymphocyte counts and reduced antibody titers to antigens (Fiske and Adams, 1985). Although not as extreme as those examples, reduced immune organ weights observed with in utero heat stress may be associated with slowed fetal growth and compromised immune function.

Consistent with the overall effect of in utero heat stress on BW, there was an effect of in utero heat stress on immune organ weight, such that in utero HT calves had lower thymus and spleen weights compared with in utero CL calves. This decrease in immune organ weight may result in compromised postnatal immune function, as significant functional outputs of the adaptive immune system are generated in bone marrow and the thymus (Abbas et al., 2011). In addition, maturation of T cells occurs in central lymphoid organs such as the thymus (Abbas et al., 2011). Likewise, foreign antigens and pathogens are most likely to elicit major immune responses in the spleen. Therefore, decreased thymus and spleen weights in in utero HT calves are likely to delay development of immune function in those offspring postnatally relative to CL calves. Further, calves born to and fed with colostrum from cows exposed to heat stress during late gestation have compromised passive immunity compared with calves born to cows that were cooled. Specifically, calves exposed to heat stress in utero had decreased AEA and total IgG concentration in serum during the first 28 d of age compared with those cooled in utero (Tao et al., 2012). This response is due to reduced IgG uptake in the small intestine of the calf rather than the colostrum consumed (Monteiro et al., 2014); when in utero heat-stressed calves consume colostrum from a single pool rather than that from their own dam, they still had decreased AEA in serum during the first 28 d of age compared with calves that were cooled in utero. Thus, decreased IgG in serum may initially result from reduced uptake from colostrum but later be related to the lower thymus and spleen weights in in utero heat-stressed calves.

Following colostrum ingestion, macromolecules such as immunoglobulins are absorbed at the intestine through a neonatal Fc receptor-independent and nonselective process (Baintner and Kocsis, 1984), and the process is mediated by “transport vacuoles,” as reviewed in Baintner (2007). Transported antibodies are released into the lamina propria before absorption into the lymphatic or portal circulation. It appears that the loss of absorptive capacity, which declines after about 6 h and is gone by 48 h of life, results from rapid turnover of enterocytes at that same time (Staley and Bush, 1985; Sangild, 2003). Of interest, Castro-Alonso et al. (2008) observed that IgG absorption is associated with enterocyte apoptosis in goat kids, further evidence that loss of the initial population of intestinal cells may be associated with antibody uptake. Thus, delaying apoptosis may improve the success of passive immune transfer. Indeed, the observation that heat-stressed calves have higher rates of jejunal cell apoptosis is consistent with more rapid gut closure and reduced ability to absorb colostral IgG. Specifically, our data indicate that jejunal cell apoptosis decreases with time after birth, mirroring gut closure. This outcome is consistent with work in goat kids, in which Castro-Alonso et al. (2008) suggested that IgG absorption is mediated by apoptosis of enterocytes, which are replaced by enterocytes with tight junctions resistant to movement of larger molecules such as Ig.

Colostrum feeding may also affect the timing of gut closure. David et al. (2003) compared ileal Peyer's patch apoptotic rates in normal-term calves immediately after birth (i.e., no colostrum) and after feeding colostrum for 4 d. Relative to the calves that did not receive colostrum, colostrum-fed calves had a reduced number of apoptotic cells in Peyer's patches, which suggests that colostrum intake reduces apoptosis in Peyer's patches (David et al., 2003), as in humans (Srivastava and Srivastava, 1999). One limitation of the study by David et al. (2003) was the lack of a group of calves at d 5 that were not fed colostrum, so the exact nature of the effect of colostrum on apoptosis cannot be determined. However, our observation that the apoptotic rate in the jejunum of calves is greater before colostrum feeding than after colostrum feeding is consistent with colostrum intake reducing the progression of apoptosis.

In conclusion, the substantial difference in heat strain on HT versus CL cows during late gestation significantly affected overall growth in utero, fetal primary lymphoid tissue development, and intestinal apoptosis. Late-gestation heat stress increases jejunal apoptosis in the first 2 d of life, indicating that in utero heat stress may accelerate gut closure relative to CL and is associated with decreased IgG uptake and limited passive immune competence. Consequently, decreased IgG uptake and reduced immune function following gestational heat stress may lead to reduced health and growth of the calf in the long term. Of particular concern from a management perspective is the observation that this acceleration of gut closure appears to occur even before birth and before colostrum consumption. Thus, it may be challenging to reverse after parturition, so management efforts should focus on cooling the dam during late gestation.
